# Factors associated with short-term and long-term mortality in solid cancer patients admitted to the ICU

**DOI:** 10.1186/cc14620

**Published:** 2015-03-16

**Authors:** R Fisher, C Dangoisse, S Crichton, S Slanova, L Starsmore, T Manickavasagar, C Whiteley, M Ostermann

**Affiliations:** 1 Guy's and St. Thomas' NHS Trust, London, UK; 2 King's College London, UK

## Introduction

Despite multiple reports demonstrating an improvement in outcomes of critically ill cancer patients admitted to ICUs over the last two decades [1], there is concern that admission policies for patients with cancer are overly restrictive [2]. The purpose of this study was to identify factors associated with mortality in the 180 days following unplanned ICU admission in patients with nonhaematological malignancy.

## Methods

We carried out a retrospective analysis of all patients with solid tumours admitted to the Guy's Critical Care Unit (13-bed level 3 ICU) as an emergency between August 2008 and July 2012. Data were collected regarding patient demographics, type of cancer, reason for referral and organ support required during the ICU stay.

## Results

During the 4-year study period there were 356 unplanned admissions of patients with solid cancer (8.3% of all admissions). Three hundred individual patients were admitted and 180-day survival data were available for 293 of these. Mean age at first admission was 65.2 years, 115 (38.3%) were female. The most frequently present malignancies were lung (42.7%), head and neck (17.3%) and renal (6.7%). ICU, hospital and 180-day mortality were 19.1%, 31.0% and 52.2% respectively. Those factors found to be independently associated (in multivariate analysis) with increased risk of 180-day mortality include: metastases (OR = 4.0, 95% CI = 2.1 to 7.6); sepsis on admission (OR = 2.2, 95% CI = 1.2 to 4.1); APACHE II score on admission (OR = 1.06, 95% CI = 1.004 to 1.12); need for inotropes/vasopressors during admission (OR = 2.3, 95% CI = 1.05 to 4.8); and need for renal replacement therapy during admission (OR = 4.65, 1.7 to 12.8).

## Conclusion

In our study, ICU and hospital mortality were lower than the pooled mortalities seen in a recent large systematic review [3] - despite our study excluding planned postoperative admissions (who are known to have better outcomes). The 180-day mortality was significantly lower than in a previous study at our own institution [4]. Our study looked at a number of factors that might reasonably be expected to be associated with short-term and long-term outcomes and identified several that were independent predictors of death by 180 days.
